# Targeted immune epitope prediction to HHLA2 and MAGEB5 protein variants as therapeutic approach to related viral diseases

**DOI:** 10.1186/s12865-021-00440-w

**Published:** 2021-07-28

**Authors:** Daniel A. Achinko, Anton Dormer, Mahesh Narayanan, Elton F. Norman

**Affiliations:** PepVax, Inc., 0411 Motor City Drive, Suite #750, Bethesda, MD 20817 USA

**Keywords:** MAGEB5, HLA, HHLA2, Immunotherapy, Epitopes, Viral-oncology

## Abstract

**Background:**

Targeted immunotherapy is mostly associated with cancer treatment wherein designed molecules engage signaling pathways and mutant proteins critical to the survival of the cell. One of several genetic approaches is the use of in silico methods to develop immune epitopes targeting specific antigenic regions on related mutant proteins. In a recent study we showed a functional association between the gamma retrovirus HERV-H Long Terminal Associating (HHLA1, HHLA2 and HHLA3) proteins and melanoma associated antigen of the B class proteins (MAGEB5), with a resultant decrease in expression of HLA class I and II immune variants. HLA-C and HLA-DRB5 were the main HLA class I and II Immune variants, respectively, that showed expression changes across viral samples of interest. Specific immune variants for HLA-C and HLA-DRB5 were filtered for the top ten based on their relative frequency of counts across the samples.

**Results:**

Protein variants for HHLA1, HHLA2, HHLA3 and MAGEB5 were used to predict antigenic epitope peptides to immune peptide-MHC class I and II binding using artificial neural networks. For IC50 peptide scores (PS) ≥ 0.5 with a transformed binding ability between 0 and 1, the top 5 epitopes identified for all targeted genes HHLA1,2 & 3 and MAGEB5 were qualified as strong or weak binders according to the threshold. Domain analysis using NCBI Conserved Domain Database (CDD) identified HHLA2 with immunoglobulin-like domains (Ig_C1-set) and MAGEB5 with the MAGE Homology Domain (MHD). Linear regression showed a statistical correlation (*P* < 0.001) for HHLA2 and MAGEB5 predicted epitope peptides to HLA-C but not HLA-DRB5. The prediction model identified HLA-C variant 9 (HLA-C9, BAA08825.1 HLA-B*1511) at 1.1% as the most valuable immune target for clinical considerations. Identification of the 9-mer epitope peptide within the domain showed for HHLA2: YANRTSLFY (PS = 0.5837) and VLAYYLSSSQNTIIN (PS = 0.77) for HLA-C and HLA-DRB5, respectively and for MAGEB5, peptides: FVRLTYLEY (PS = 0.5293) and YPAHYQFLWGPRAYT (PS = 0.62) for HLA-C and HLA-DRB5, respectively.

**Conclusion:**

Specific immune responses to targeted epitope peptides and their prediction models, suggested co-expression and co-evolution for HHLA2 and MAGEB5 in viral related diseases. HHLA2 and MAGEB5 could be considered markers for virus related tumors and targeted therapy for oncogenic diseases.

**Supplementary Information:**

The online version contains supplementary material available at 10.1186/s12865-021-00440-w.

## Introduction

Epitopes are molecular structures recognized by immune receptors as targets [1]. Binding epitopes are presented to CD8+ and CD4+ T cells by class I and class II MHC molecules, respectively. Binding affinity of epitopes to the different MHC molecules is very important in determining immunogenicity [2]. Every MHC molecule has a potential uniqueness specific to its binding ability to a distinct set of antigenic peptides [3]. There has been a great advancement in the development of algorithms that identify peptide regions in targeted genes against immune cells and they focus on binding affinity prediction of known peptides [4]. MHC peptide epitopes with high binding affinity have been associated with strong immune responses and though the necessity of high binding affinity of the peptide, it is not sufficient to qualify immunogenicity [5]. The NetMHCpan method [6] uses Artificial Neural Networks (ANN) as a method for peptide prediction. ANN is ideally the most recognized method to identify non-linear patterns believed to be part of HLA-I interactions [7]. NetMHCpan exploits both peptide and the primary HLA sequence as the information inputted to drive ANN predictions, which incorporates all known and available HLA variant data and the method output was presented in units of predicted affinity (IC_50_ nM) [8].

The Human Endogenous Retrovirus-H Long Terminal Repeat (HERV-H LTR) are retroviral sequences that are integrated into the genome in the course of evolution and are known to cover 8% of the human genome [9]. HERVs are also known as retrotransposons due to absence of the envelope gene. Their condition for functional transcription is that HERV sequences should maintain an LTR that is functional or controlled by a different promoter not affected by nucleotide substitutions or deletions interrupting the open reading frames (ORFs) [10]. Retroviruses integrated primate genome via exogenous infection, which could affect somatic and at times germline cells resulting in their vertical transmission to the offspring in a Mendelian fashion with a fix in the human population [11, 12]. Envelope retroviruses, HERV-W and HERV-K are shown to be involved in Multiple Sclerosis (MS) and Amyotropic Lateral Sclerosis (ALS), respectively and are diseases of the nervous system [13, 14]. HERV-H are non-envelope viruses and derive three genes known as: HHLA1 with cytogenic location 8q24.22, HHLA2 with genomic location 3q13.13 and HHLA3 with genomic location 1p31.1. Work done by [15] identified from the EST database, 2 genes (HHLA2 and HHLA3) which had transcripts poly adenylated with an LTR of the HERV-H family.

HHLA2 is part of the B7 family and a co-stimulatory molecule with a role in activating and downregulating T lymphocytes. It has distinct genomic properties in that it expresses 3 immunoglobulin (Ig) domains in the extracellular region of the protein. HHLA2 specifically binds CD28H and co-stimulates T cells [16]. In the immune system, HHLA2 protein expresses in a combined fashion on monocytes and dendritic cells and upregulated by inflammatory signals such as lipopolysaccharide and IFNy. HHLA2 has not been shown to express on resting T or B cells but upregulated when T cells are activated [17]. HHLA2 is expressed in about 20 to 70% of large number of human cancers including lung, thyroid, breast, pancreas, melanoma, bladder, colon just to name a few [18]. This work further showed that HHLA2 was highly expressed in about 50% of tumors related triple-negative breast cancer (TNBC) with a high risk of spreading. The expression of Melanoma associated antigens (MAGE) have also been shown to be highly expressed in TNBC but co-expression of HHLA2 and MAGE related genes especially MAGEB5 have not been demonstrated in any cancer type.

The family of MAGE genes comprises 19 members and located on chromosome X. These genes further subdivide into four families (A to D) which is based on their chromosomal location and observed similarities between encoded proteins. There are four known MAGEB genes located on Xp21.3 [19]. MAGE A, B and C genes have not been shown to express in normal tissues except in the testes. MAGEB5 and MAGEB6 have been identified in different tumor types and the activation of these genes stems from the demethylation of their relative promoter regions [20]. Work done by [20, 21] didn’t locate MAGEB5 in the testes but identified it in a seminoma, which is a malignant neoplasm of the testes with a 95% treatment success when discovered early. The expression pattern of MAGEB5 given its absence in the testes may suggest its specificity of co-expression with HHLA2 in viral cancer related diseases and tumors.

Given the plethora of MHC polymorphisms which specifically identify a subset of peptides facilitating cellular immunity and also providing broad coverage of alleles, makes it an essential task yet complicated for vaccine discovery [22]. Modeling immune variants and genetic interactions related to cancer has not been done before and our work shows a prediction model in which all immune variants predicting epitope peptides for co-expressed genes could be considered for identifying immune variants with potential clinical outcomes for vaccine development.

HHLA2 and MAGEB5 have been individually shown to be expressed in various tumor types and the former has been associated with viral related diseases. No data online showed an investigated analysis of co-expression pattern between HHLA genes with focus on HHLA2 and MAGE genes with focus on MAGEB5. The observed co-expression pattern between HHLA and MAGEB5 as demonstrated by our previous article [23] and this one, are the first to show co-expression and co-regulated patterns which can occur independently in viral and cancer diseases and also in viral related cancer diseases.

## Materials and methods

### Data used

**Fig. 1:**
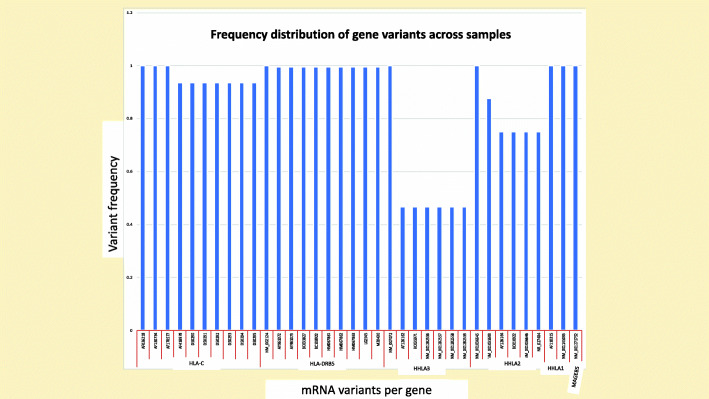
Frequency Distribution of Immune and Protein Related Gene Variants: This figure shows the frequency distribution of the various protein variants used in the study. The top ten immune variants per immune gene (HLA-C and HLA-DRB5) were considered for this study and all peptide variants identified for HHLA1 (*n* = 2), HHLA2 (*n* = 7), HHLA3 (*n* = 5) and MAGEB5 (*n* = 1) were all considered for this study. HHLA3 had protein variants with the lowest frequency

This work is based on further analysis of data obtained in our last article [23]. The datasets generated and analyzed during the current study were obtained from the GEO (RRID: SCR_005012) repository, and specifically GEO Profiles database (www.ncbi.nlm.nih.gov/geoprofiles/) with the Accession numbers: GDS5093, GDS4424, GDS5614, GDS5613, GDS2606, GDS4238, GDS2023, GDS3489, GDS2676, GDS4669. The data showed that, variants of melanoma associated antigens (MAGE) of the B class (MAGEB5) protein, genetically coevolved with the Human Endogenous Retrovirus-H Long Terminal Repeat-Associating 2 (HERV-H LTR-A2) gene (HHLA2). HHLA2 protein variant encodes a protein found on B-cells (monocytes) and known to regulate cell immunity by binding to a particular site on T-lymphocytes with aim to inhibit their proliferation. MAGEB5 identified with one nucleotide variant (NM_001271752) (Dataset 1) and HHLA2 identified with 7 nucleotide variants (AF126162, BC035971, NM_001282556, NM_001282557, NM_001282558, NM_001282559) (Dataset 2) but the most abundant was, NM_007072. Given that HHLA1 and HHLA3 are members of the HHLA gene cluster and were shown to co-express together, they were also considered for epitope analysis in this article. HHLA1 identified with 2 variants (AF110315, NM_001145095) (Dataset 3) of the same abundance and HHLA2 identified with 6 variants (NM_001031693, AF126164, BC010922, NM_001036646, NR_027404) (Dataset 4) with the most abundant being NM_001036645. Two immune gene variants, HLA-C and HLA-DBR5 for HLA class I and HLA class II, respectively showed a considerable expression level across samples and were considered for further analysis in this study. Majority of the variants for HLA-C (Dataset 5) and HLA-DBR5 (Dataset 6) had high abundance in the data but only the top 10 variants for HLA-C (AF026218, AF130734, AF170577, AF418978, D50290, D50291, D50292, D50293, D50294, D50295) and HLA-DRB5 (NM_002124, AY961072, AY961073, BC033827, BC108922, HM067861, HM067862, HM067863, L02545, M20430), were considered. The graphical distribution of all the gene related variants for this study is seen on Fig. 1.

### HLA class I epitope prediction for MAGEB5 and HHLA 1, 2, & 3 variants

The protein sequences in FASTA format were obtained for all identified HLA-C, HHLA1, HHLA2, HHLA3 and MAGEB5 variants from the National Center of Biotechnology Information (NCBI) Protein database. The NetMHCpan 4.0 Server [24] based on prediction of peptide-MHC class I binding using artificial neural networks (ANN) was used to predict binding peptide epitopes to MHC. The method was trained on ligands naturally eluted alongside binding affinity data. The server uses neural networks to predict the binding to any MHC molecule of a known targeted peptide of interest. Our targets of interest were protein variants for HHLA 1, 2 &3 and MAGEB5. Each targeted sequence was individually matched to each HLA-C protein variant and identified epitope peptide targets of 9 amino acids sequence in length were classified as strong binders at a %Rank < 0.5 and weak binders at %Rank > 2. Screening for immune peptides was based on strong binders and given that no strong binders were identified, the top 5 predicted peptide epitopes for all HLA-C protein variant were considered for further analysis using the IC50 value also known as the binding affinity value of the MHC to the peptide at a given concentration. The range between 0 and 1 defined a negative binder to the MHC = 0 and a positive binder = 1.

### HLA class II epitope prediction for MAGEB5 and HHLA 1, 2, & 3 variants

Protein sequence variants for HLA-DR5, HHLA1, HHLA2, HHLA3 and MAGEB5 in FASTA format were obtained from the National Center of Biotechnology Information (NCBI) Protein database. The NetMHCIIpan 3.0 Server [25] based on prediction of binding peptides for MHC class I was used to predict epitopes to targeted proteins of interest which were protein variants for HHLA 1,2 & 3 and MAGEB5. Each targeted protein sequence was individually matched to each HLA-DR5 protein immune variant and identified epitope peptide of 15 amino acids in sequence length were classified as strong binders at a %Rank < 2 and weak binders at %Rank > 10. Screening for immune peptides was based on strong binders and given that no strong binders were identified, the top 5 predicted peptide epitopes for each HLA-DR5 protein variant were considered for further analysis using the IC50 value also known as the binding affinity value of the MHC to the peptide on the targeted protein at a given concentration. The range between 0 and 1 defined a negative binder to the MHC = 0 and a positive binder = 1. Characteristic graphical representations of each amino acid on the predicted epitope was obtained based on binding score of each amino acid on the sequence. This acted as a guide to core peptides on the string.

### Protein variant and domain analysis

Protein conformation is highly dependent on domain type and critical to the prediction of antigenic sites for immune detection and destruction. Domain prediction on the targeted protein variants of related genes (HHLA 1,2 & 3 and MAGEB5) was done through the Conserved Domain Database (CDD) which is a protein annotation source consisting of multiple sequences aligned using models related to ancient domains and proteins of full length [26].

### Statistical analyses

Selection of MHC HLA class I (HLA-C) and II (HLA-DRB5) immune variants for epitope prediction analysis was based on all identified variants per sample from previous work [23]. The variants with highest abundance in the data were based on the proportion of each variant per immune gene across all samples divided by the sum of total variants for the given gene (ni/nT, I = individual variant, T = total gene variants) across all samples in the dataset. The selection of top 5 peptides per protein variant for targeted genes (HHLA 1, 2 & 3 and MAGEB5) was based on highest peptide score (≥ 0.5), known as log transformed binding affinity (aff) values measured as IC50 in nM with formula 1- log50k(aff) in the range of 0–1 (Jensen et al., 2018). Two peptide regions were detected within the dataset as core and icore peptides. The core peptides were 9 AA regions predicted in direct contact with MHC and suggested mutation sites while the icore defined immune protein interaction of peptide core of actual protein sequence subject to insertions and deletions. This makes the targeted region of great clinical importance when detecting for genetic variance within a said population and possible resistance to designed medication. Linear regression analysis between targeted genes (HHLA2 and MAGEB5) was done using Wizard statistical software version 1.9.34 [27] with a 95% confidence interval and data modeling of each immune gene, HLA-C and HLA-DRB5, and its variants as outcome variable against HHLA2 and MAGEB5 as dependent variables was also performed using formula: Immune gene = HHLA2 + MAGEB5 + C (constant). The null hypothesis was considered at β ≤ 0 for statistical significance consideration which defined the coefficients of the dependent variables in the model. The post analysis of predicted peptide epitopes data considered three parameters: i) the protein variant type, ii) the epitope peptide variant per protein and iii) the peptide score.

A) The prediction of a single but different antigenic epitope peptides with different scores for different protein variants of the same gene: This indicates the prediction by the same immune variant to different antigenic regions of protein variants of the same gene. The prediction of several but different singular epitope peptides for the same or different antigenic  protein variants of the same gene. This indicates the peptide prediction by the same or different immune variants to the protein variants of the same gene.

**Table:** 

Protein variant	Peptide epitope	score
Variant A	PHDSTAGKG FGIHKLMQR	0.70 0.69
Variant B	RFKHYDEDY ADELIRNGG	0.80 0.75

B) The prediction of a single but the same antigenic epitope peptide with the same score for different protein variants of the same gene: This indicates the peptide prediction by the same immune variant to different protein variants of the same gene.

**Table:** 

Protein variant	Peptide epitope	score
Variant A	PHDSTCGK	0.7
Variant B	PHDSTCGK	0.7

III) The prediction of the same antigenic epitope peptide for different protein variants of the same gene presenting with different scores: This indicates the prediction by different immune variants to the same peptide region of different protein variants of the same gene.

**Table:** 

Protein variant	Peptide epitope	score
Variant A	PHDSTCGKG	0.70
Variant B	PHDSTCGKG	0.60

IV) The prediction of the same antigenic epitope peptide repeatedly for a given protein variant of the same gene having different scores: This indicates that the predicted peptide region was identified by different immune epitopes for the same protein variant of a given gene.

**Table:** 

Protein variant	Peptide epitope	score
Variant A	PHDSTCGKG PHDSTCGKG	0.70 0.65
Variant B	RFGHYDEDY RFGHYDEDY	0.60 0.58

E) The prediction of a similar antigenic epitope peptide region with single nuclear variation for a gene having a different score: This indicates that the same or different immune protein variants predicted at the same immunogenic site, different variants for the same antigenic epitope peptide in the targeted protein variant of a given gene. This mutant peptide could be favored by translocation which is a property of virus-related proteins.

**Table:** 

Protein variant	Peptide epitope	score
Variant A	PHDST**A**GKG PHDST**C**GKG	0.70 0.65
Variant B	RF**K**HYDEDY RF**G**HYDEDY	0.60 0.58

## Results

### MHC HLA class I predicted binding peptide epitopes to HHLA 1, 2 & 3 and MAGEB5

**Fig. 2:**
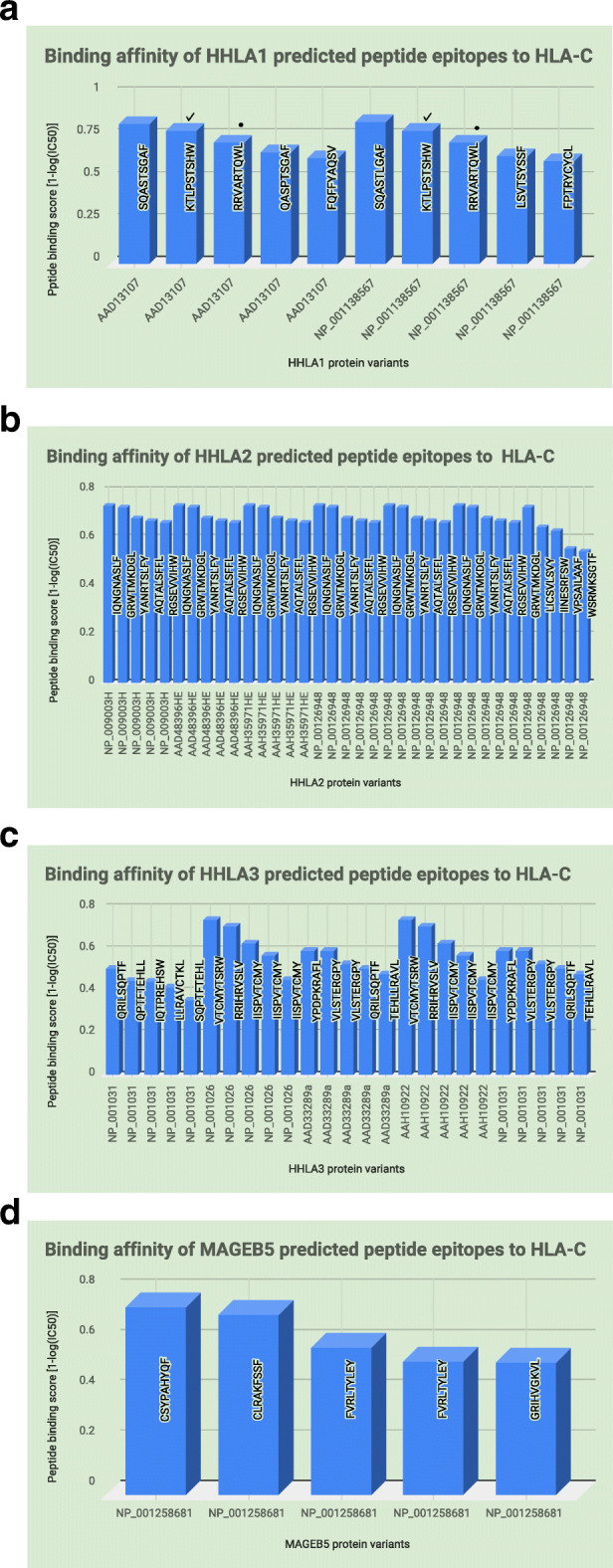
Binding Affinity of Predicted Epitope Peptides to Target Genes (HHLA1,2 & 3 and MAGEB5) by HLA-C Immune variants: Each Immune variant was used at the NetMHCpan 4.0 Server to predict epitope peptides for each protein variant of the target genes. The plotted graphs showed gene-protein variants on the x-axis against and their different predicted epitope peptides represented by their peptide binding score on the y-axis and measured by their binding affinity; 1-log (IC50). **A** This figure shows the number of protein variants for HHLA1 (*n* = 2) and number of predicted unique epitope peptides (*n* = 8). Two epitope peptides [KTLPSTSHW (check) & RRVARTQWL (dot)] were common to both protein variants and one epitope peptide (QASPTSGAF) was predicted for amino acid variations. **B** This figure shows the number of protein variants for HHLA2 (*n* = 7) and number of unique predicted epitope peptides (*n* = 9) with 5 of them (AQTALSFFL, GRWTMKDGL, IQNGNASLF, RGSEVVIHW, YANRTSLFY) repeating in almost all protein variants. **C** This figure shows the number of protein variants for HHLA3 (*n* = 5) and number of unique predicted epitope peptides (*n* = 11) with 7 of them (IISPVTCMY, QRILSQPTF, RRIHRVSLV, TEHLLRAVL, VLSTERGPY, VTCMYTSRW, YPDPKRAFL) repeating in at least two protein variants. **D** This figure shows the number of protein variants for MAGEB5 (*n* = 1) and number of unique predicted epitope peptides (*n* = 4) with one (FVRLTYLEY) repeating due to different binding scores

The top 5 predicted peptide epitopes to HLA-C obtained for each targeted gene variant showed a differential sequence pattern to the various immune variants used for their prediction. For HHLA1, two predicted epitope peptide variants were common across (KTLPSTSHW & RRVARTQWL) with the same peptide score meanwhile another epitope peptide showed mutational changes (SQAST**L**GAF, SQAST**S**GAF, QAS**P**T**S**GAF, blue = unchanged nucleotides, red = mutations) with different scores across protein variants of the gene (Fig. 2A). For HHLA2 (Fig. 2B), one predicted epitope peptide (GRWTMKDGL) was common for all 7 protein variants with the same peptide score and 6 peptide epitopes (IQNGNASLF, YANRTSLFY, AQTALSFFL, RGSEVVIHW) were common for 6 protein variants with the same score. For HHLA3 (Fig. 2C), one epitope peptide (IISPVTCMY) was predicted 3 times each for two protein variants. The peptide scores were different per protein variant but the same for each peptide for the different protein variants. The peptide VLSTERGPY was predicted twice for two protein variants. The peptide scores were different per protein variant but the same for each peptide for the different protein variants. The peptides QRILSQPTF and RRIHRVSLV were predicted 3 and 2 times for three and two protein variants, respectively. The peptide scores were different per protein variant but the same for each peptide for the different protein variants. For MAGEB5 (Fig. 2D), FVRLTYLEY was the only predicted peptide that occurred twice with different scores, for the only identified protein variant for this gene. All other peptides were different and with different scores.

### MHC HLA class II predicted binding peptide epitopes to HHLA 1, 2 & 3 and MAGEB5

**Fig. 3:**
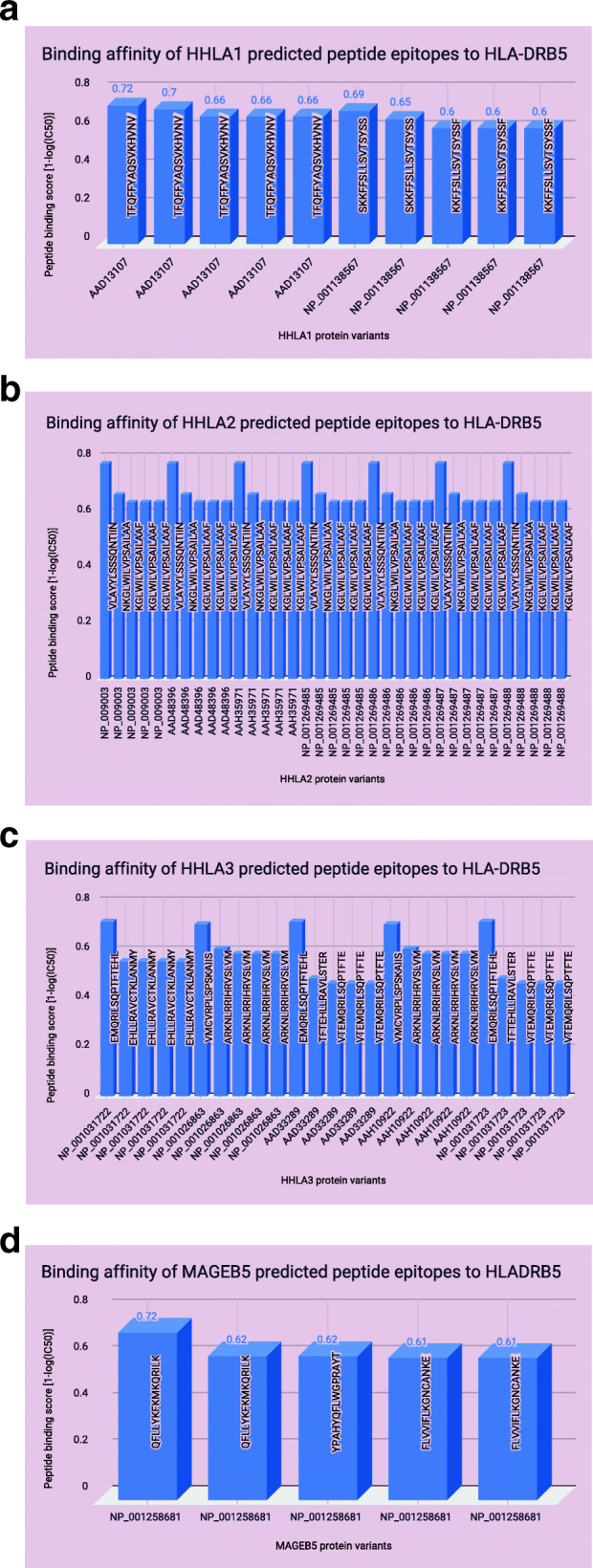
Binding Affinity of Predicted Epitope Peptides to Target Genes (HHLA1,2 & 3 and MAGEB5) by HLA-DRB5 Immune variants: Each Immune variant was used at the NetMHCIIpan 3.0 to predict epitope peptides for each protein variant of the target genes. The plotted graphs showed gene-protein variants on the x-axis against and their different predicted epitope peptides represented by their peptide binding score on the y-axis and measured by their binding affinity; 1-log (IC50). **A** This figure shows the number of protein variants for HHLA1 (*n* = 2) and number of predicted unique epitope peptides (*n* = 3). The three epitope peptides were common to both protein variants. **B** This figure shows the number of protein variants for HHLA2 (*n* = 7) and number of unique predicted epitope peptides (*n* = 3) repeating in all protein variants. **C** This figure shows the number of protein variants for HHLA3 (*n* = 5) and number of unique predicted epitope peptides (*n* = 6) repeating in at least two protein variants. **D** This figure shows the number of protein variants for MAGEB5 (*n* = 1) and number of unique predicted epitope peptides (*n* = 3) with two of them (FLVVIFLKGNCANKE & QFLLYKFKMKQRILK) repeating due to different binding scores

HLA-DRB5 was the targeted MHC class II immune variant used to predict 15 mer epitopes to HHLA (1,2 & 3) and MAGEB5 genes. For HHLA1 (Fig. 3A), two different peptides TFQFFYAQSVKHVNV and SKKFFSLLSVTSYSS were identified for the two protein variants with three different peptide scores. For HHLA2 (Fig. 3B), one peptide (VLAYYLSSSQNTIIN) was predicted with the highest score for all protein variants, then epitope peptide NKGLWILVPSAILAA was predicted with next highest score across all protein variants, then epitope peptide KGLWILVPSAILAAF was predicted three times for each protein variant with the same score across all protein variants for the gene. For HHLA3 (Fig. 3C), epitope peptide EMQRILSQPTFTEHL was predicted with the same score for 3 different protein variants, then VMCVRPLSPSKAIIS was predicted with the same peptide score for 2 protein variants, then epitope peptide ARKNLRRIHRVSLVM was predicted four times two different scores per protein variant and this pattern was the same for another protein variant. For MAGEB5 (Fig. 3D), epitope peptide QFLLYKFKMKQRILK and FLVVIFLKGNCANKE were predicted two times for the same protein variant but the former had different peptide scores while the latter had the same peptide score.

### Predicted peptide domain analysis

**Fig. 4:**
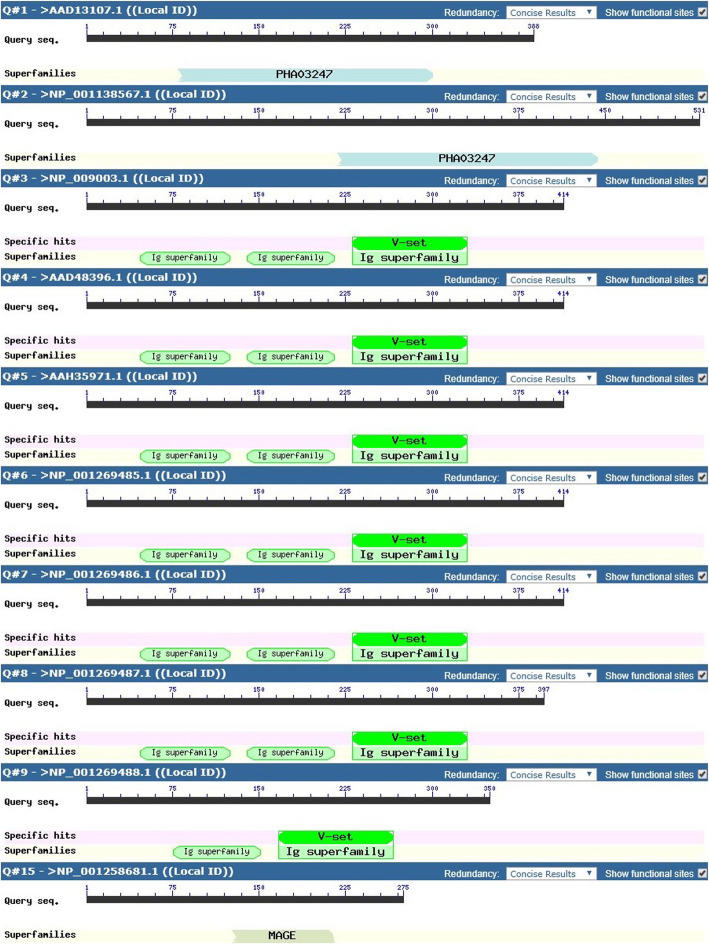
Domain Predictions for Protein Variants of Targeted Genes (HHLA1,2 & 3 and MAGEB5). This figure shows the different domains identified for the targeted protein variants of related genes (HHLA1,2 & 3 and MAGEB5). The superfamily Polyhydroxyalkanoates domain (PHA03247) was identified for the two protein variants for HHLA1 though at different amino acid positions. Six out of the 7 protein variants for HHLA2 demonstrated Immunoglobulin V-set domain (pfam07686) common to antibodies and three superfamily Immunoglobulin C1-set domains (pfam07654) and superfamily Immunoglobulin domains (cl11960) found in HERV-H LTR-associating 2. One HHLA2 variant identified with one Immunoglobulin V-set, one superfamily Immunoglobulin C1-set and one superfamily Immunoglobulin domains. For MAGEB5, MAGE Homology Domain (MHD) (pfam01454) was identified on the only protein variant

The four targeted genes (HHLA 1, 2 & 3, MAGEB5) and related protein variants were subjected to domain analysis and out of the 15 total variants, HHLA3 protein variants identified with no known domains (Fig. 4). For HHLA1, the superfamily Polyhydroxyalkanoates domain (PHA03247) was identified for the two protein variants though at different amino acid locations. For HHLA2, six out of the 7 protein variants identified with one Immunoglobulin V-set domain (pfam07686) found on antibodies and three superfamily Immunoglobulin C1-set domains (pfam07654) and superfamily Immunoglobulin domains (cl11960) in HERV-H LTR-associating 2, per variant was identified. One HHLA2 variant identified with one Immunoglobulin V-set, one superfamily Immunoglobulin C1-set and one superfamily Immunoglobulin domains. For MAGEB5, MAGE Homology Domain (pfam01454) was identified on the only identified protein variant. The derived epitope peptides for MHC HLA class I and II obtained for the various targeted genes were evaluated for their domain presence. For HHLA1 protein variants, two HLA Class I epitope peptides (QASPTSGAF and RRVARTQWL) and no epitope peptide for HLA class II were identified in the domain. For HHLA2 protein variants, 5 HLA class I epitope peptides (RGSEVVIHW, YANRTSLFY, IQNGNASLF, LICSVLSVY and IINESRFSW) and one epitope peptide (VLAYYLSSSQNTIIN) for HLA class II were identified in the domain. No domains were identified for HHLA3 protein variant sequences used for peptide prediction while for MAGEB5 protein variants, two HLA class I epitope peptides (FVRLTYLEY and CSYPAHYQF) and two HLA class II epitope peptides (FLVVIFLKGNCANKE and YPAHYQFLWGPRAYT) were identified in the domain.

### Linear regression and modeling of immune data

**Fig. 5:**
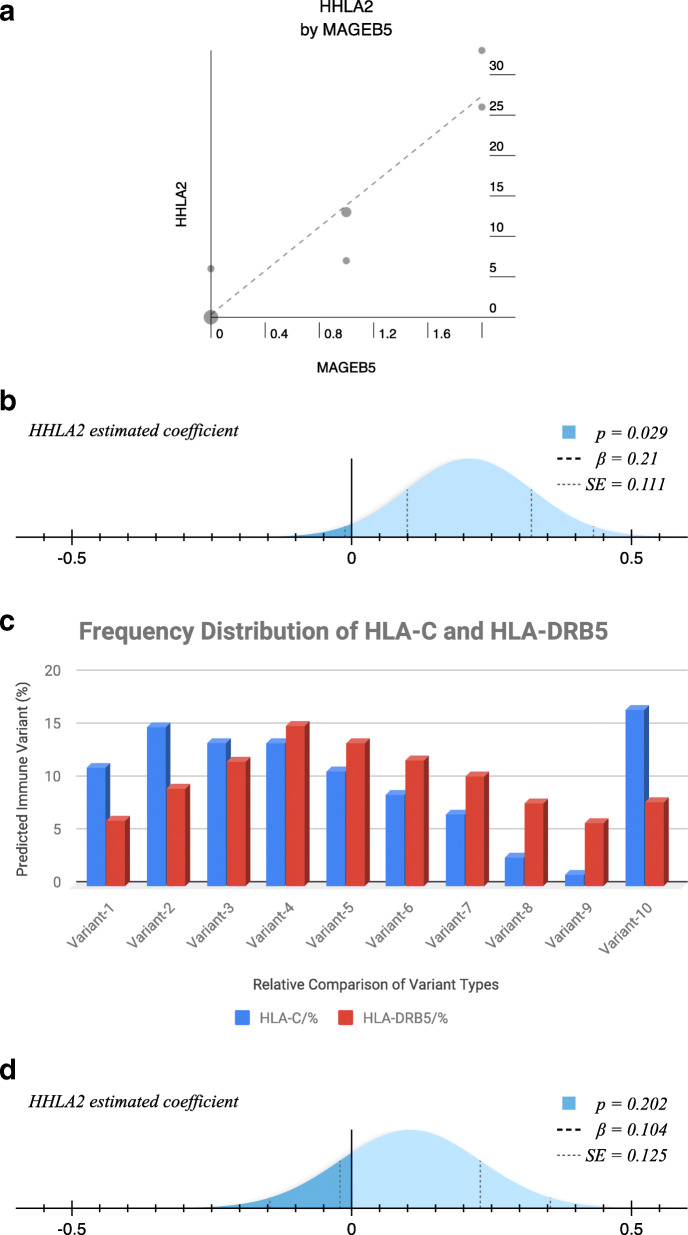
Statistical Analysis Representation of Genetic Association Between HHLA2 and MAGEB5: These figures are based on epitope prediction counts by HLA-C and HLA-DRB5 on protein variants from HHLA1, HHLA2, HHLA3 and MAGEB5. Analysis demonstrated a suggested genetic relationship between HHLA2 and MAGEB5, possible co-expression and co-evolution patterns in a given biological system. **A** This figure shows a regression analysis plot of positive correlation (Pearson correlation, *p* < 0.001) between HHLA2 and MAGEB5 epitope peptides predicted by HLA-C. **B** This figure shows the predicted model (HLA-C = HHLA2 + MAGEB5 + C), β = 0.21 (< 0), standard error, SE = 0.111 and *p* = 0.029. Only the variation of HHLA2 and the constant were statistically significant in the model. C) The predicted impact frequency of the various HLA-C immune variants used in the model were compared to those predicted HLA-DRB5. Though opposing effects were observed for relative immune variants, they weren’t comparable because they are different immune genes. D) The predicted model (HLA-DRB5 = HHLA2 + MAGEB5 + C) had the following parameters: β = 0.104, SE = 0.125 and *p* = 0.202, with equation: HLA-DRB5 = 0.104 (HHLA2) – 0.711 + 1.13. The variation for each of the targeted genes was not significantly important in impacting the model

For analysis on HLA-C predicted epitope peptides for targeted genes (HHLA 1, 2 & 3) (Table 1), there was a positive linear correlation (Pearson correlation, *p* < 0.001) observed only between HHLA2 & MAGEB5 (Fig. 5A) and for the predicted model (HLA-C = HHLA2 + MAGEB5 + C), β = 0.21 (< 0), standard error, SE = 0.111 and *p* = 0.029 (Fig. 5B). The resultant equation post analysis was HLA-C = 0.21 (HHLA2) – 2.194 (MAGEB5) + 1.218. Only the variation of HHLA2 and the constant were statistically significant in the model. The predicted impact of the various HLA-C immune variants used in the model were as follows: HLA-C1 = 11.2%, HLA-C2 = 15%, HLA-C3 = 13.5%, HLA-C4 = 13.5%, HLA-C5 = 10.9%, HLA-C6 = 8.7%, HLA-C7 = 6.8%, HLA-C8 = 2.7%, HLA-C9 = 1.1% and HLA-C10 = 16.7% (Fig. 5C). For analysis on HLA-DRB5 predicted epitope peptides for targeted genes (HHLA 1, 2 & 3) (Table 2), no positive linear correlation was observed (*p* = 0.724) and especially between HHLA2 & MAGEB5. The predicted model (HLA-DRB5 = HHLA2 + MAGEB5 + C) had the following parameters; β = 0.104, SE = 0.125 and *p* = 0.202 (Fig. 5D) with equation: HLA-DRB5 = 0.104 (HHLA2) – 0.711 + 1.13. The variation for each of the targeted genes was not significantly important in impacting the model. The predicted impact of the various HLA-DRB5 immune variants used in the model were as follows: HLA-DRB5–1 = 6.2%, HLA-DRB5–2 = 9.2%, HLA-DRB5–3 = 11.8%, HLA-DRB5–4 = 15.2%, HLA-DRB5–5 = 13.5%, HLA-DRB5–6 = 11.9%, HLA-DRB5–7 = 10.4%, HLA-DRB5–8 = 7.8%, HLA-DRB5–9 = 6% and HLA-DRB5–10 = 8% (Fig. 5C).

**Table 1:** HLA-C Immune Epitopes Count for HHLA and MAGEB5: This table presents the various counts of unique epitope peptides predicted by each HLA-C immune variant (C1–10) to protein variants for HHLA1, HHLA2, HHLA3 and MAGEB5. HHLA2 demonstrated the highest epitope peptide counts due to more protein variants (*n* = 7) while MAGEB5 had fewer epitope peptide counts due one protein variants (*n* = 1). Some immune variants never predicted any epitope peptides for any of the protein variants for the targeted proteins (HHLA 1,2 & 3 and MAGEB5)

HLA I Immune allele	HHLA1	HHLA2	HHLA3	MAGEB5
HLA-C1	2	7	7	1
HLA-C2	3	13	2	1
HLA-C3	1	0	2	0
HLA-C4	0	0	0	0
HLA-C5	1	6	0	0
HLA-C6	1	0	0	0
HLA-C7	6	26	4	2
HLA-C8	2	33	4	2
HLA-C9	1	13	0	1
HLA-C10	0	0	0	0

**Table 2:** HLA-DRB5 Immune Epitopes Count for HHLA and MAGEB5: This table presents the various counts of unique epitope peptides predicted by each HLA-DRB5 immune variant (DRB5–1-10) to protein variants for HHLA1, HHLA2, HHLA3 and MAGEB5. HHLA1 showed unique epitopes for all protein variants as predicted by all immune variants. HHLA2 demonstrated the highest epitope peptide counts due to more protein variants (*n* = 7) while MAGEB5 had fewer epitope peptide counts due one protein variants (*n* = 1). Some immune variants never predicted any epitope peptides for any of the protein variants for the targeted proteins (HHLA 1,2 & 3 and MAGEB5)

HLA II Immune allele	HHLA1	HHLA2	HHLA3	MAGEB5
HLA-DRB5–1	3	21	5	3
HLA-DRB5–2	3	14	17	2
HLA-DRB5–3	3	14	2	3
HLA-DRB5–4	3	21	5	3
HLA-DRB5–5	3	21	0	1
HLA-DRB5–6	3	21	5	3
HLA-DRB5–7	3	21	0	1
HLA-DRB5–8	3	21	3	2
HLA-DRB5–9	3	21	2	2
HLA-DRB5–10	3	21	5	3

## Discussion

This study is a follow up from a previously published work [23] on expression patterns of MAGEB5 and HHLA2 transcripts across viral and cancer associated samples, whose genetic co-expression patterns suggests viral disease and cancer causation. The up-regulation of HHLA2 and MAGEB5 (≥ 4 folds) saw a down-regulation (≥ 3 folds) of MHC HLA class I and II immune variants with a one to two-fold up-regulation of HLA-C and HLA-DRB5 for HLA class I and II immune variants, respectively. The genetic association between MAGEB5 and HHLA2 genes formed the basis for further investigating their roles for therapeutic intervention in virus and cancer related diseases. Prediction of peptide epitopes using protein variants for HLA-C and HLA-DRB5 immune genes identified several epitope peptides for targeted genes; HHLA1, HHLA2, HHLA3 and MAGEB5. The wide variation in predicted epitope peptides for targeted genes to HLA-C immune variants suggest a possible immune selection pressure dominant for HLA class I immune variants which may result from the detection of smaller antigenic regions (9 mer) compared to a larger antigenic region (15 mer) for HLA-DRB5. From previous work done by [28], it was shown that the most efficient peptide size detected by HLA class I immune molecules is 9 amino acids. Work done on peptide binding groove for HLA class I and II molecules showed that, the latter had a wider groove permitting it to cleave a longer antigenic peptide than the former [6]. The difference in peptide binding groove structure could account for organism and antigenic specificity and it has also been documented that HLA class I immune molecules are more specific to viral peptide detection [29]. The functional specificity attached to HLA class I and II immune molecules could suggest that the latter will focus on more conserved regions across different pathogens making it less focus on evolutionary spots when detecting peptide regions unlike the former which is more focused on most antigenic regions which could be subject to high antigenic variation. Our data showed a conserved detection of epitope peptides for HLA-DRB5 immune variants across targeted genes as seen in Table 2 which was not the case HLA class I epitope peptides as seen by a wide variation for HLA-C immune variants with most of them not able to identify any peptide. The most diverse epitope peptides were identified for HHLA2 which was also associated with immune-like domains. We are suggesting that the specificity of HLA class I immune variants could be the driving force for evolutionary diversity in this immune class which was observed through the predicted synonymous and non-synonymous amino acid changes for epitope peptides of HHLA1. The most diversity and number of predicted epitope peptides also occurred for the HLA class I immune class differentially detecting at least 9 peptides across the protein variants. For HLA class II immune molecules, their predicted epitope peptides were mostly the same across all the protein variants for each gene and for each HLA-DRB5 immune variants. MHC peptide epitopes with high binding affinity have been associated with strong immune responses and though the necessity of high binding affinity of the peptide, it is not sufficient to qualify immunogenicity [5]. In the same study, the quest to know if predicted epitope peptides for different selected HLA class I immune alleles had binding affinities at the threshold of 500 nM (IC50 ≤ 500 nM) using binding assays, showed that predicted epitope peptides varied relative to each immune variant with similar results for assay experiments at binding scores of 0.66 to 1.5. This information supports our selection of the epitope peptides with highest binding scores per protein variant for each gene. The prediction model determined peptide sites known as the core, which were prone to synonymous or nonsynonymous amino acid changes and this was observed only for HHLA1 gene. Core predicted epitope peptides were prone to high peptide binding scores which varied based on amino acid changes relative to the immune variant which suggested that immunogenicity of a peptide depended on the amino acid type making it critical for immune response and vaccine design [30]. HHLA 1, 2 & 3 are considered to be derived from Human endogenous retroviruses (HERV-H) made of repetitive genomic elements resulting from ancient retroviral and germline infections due to multiple viral infections. HHLA1 is considered a spliced transcript from the promoter region of HERV-H and the LTR site of HERV-H is also known to provide polyadenylation signals to HHLA2 and HHLA3 [31]. This variable expression pattern in the HHLA 1, 2 & 3 genes could be seen in the differential domain expression patterns observed. Only HHLA2 showed immune related domains with a potential to interact with immune proteins and considered an immune checkpoint protein target for therapeutic development. The protein variants for HHLA3 didn’t identify with any known domains while HHLA1 protein variants showed differential locations of the tegument protein domain common to herpes simplex virus (HSV). MAGEB5 identified with MAGE Homology domain (MHD) which has no define function yet. For all the epitope peptides predicted for HLA-C immune variants, majority were identified in the domain while those for HLA-DRB5 immune variants were identified outside the domain. The domain is considered independent functional regions of a protein with high restriction to evolutionary changes [32] which could be the preferred target site for HLA class I immune variants. The prediction of a HHLA1 epitope peptide within its identified domain and which is subject to amino acid changes suggest that amino acid variations could result from immune pressure which is used as a route to immune escape. Work done on T-cell immune targeting of HIV 1 virus showed that selective immune pressure could lead to viral escape of mutations within targeted epitopes during an acute infection [33] or in the course of a chronic infection [34]. Coevolution of genes has always been a functionally related concept and given the domain diversity for the targeted proteins, this concept was considered for HHLA2 and MAGEB5, previously shown to genetically interact [23]. MAGE genes are known to be naturally expressed only in the testis and differentially expressed in other tissues in relation to carcinogenesis [28] and just like HERV-H genes, it was shown that they are epigenetically regulated for them to be expressed. Retroviral genes have been documented to be highly methylated in their natural tissues of expression but an increase in transcript levels is observed in tumors due to hypomethylation [35] likewise for MAGE genes their promoter regions are highly methylated for epigenetic regulation but the expression of their transcripts in tumors are due to unmethylation [36]. This epigenetic regulation pattern could implicate similar transcription factors to the activation of these genes at differential locations in the genome leading them to co-express in related cancer types. From the previous work on virus and cancer data we observed the expression pattern for HHLA2 and MAGEB5 across all samples suggesting that they could co-express and co-function together. The modeling of our data considered HHLA2 and MAGEB5 as dependent variables to HLA-C and HLA-DRB5 immune variants in gene regulated and prediction outcome analysis. This type of analysis was done for its first time for MHC immune molecules and targeted epitope peptides. The relationship between HHLA2 and MAGEB5 showed a positive correlation and their modelled relationship with HLA-C was statistically significant based on HLA-C and HHLA2 relative peptide variation. The modelled relationship for HLA-DRB5 showed no positive correlation between HHLA2 and MAGEB5 and the relationship of HLA-DRB5 = HHLA2 + MAGEB5 + C showed no statistical significance based on the variation of any of the dependent variables (HHLA2 and MAGEB5). This observation suggest that the differential selection of epitope peptides is a critical relationship between the immune variants for HLA class I (HLA-C) immune variants and the related gene of interest which is HHLA2 for this study. The statistical significance showed a percentage prediction of the immune variant whose interaction with HHLA2 will inhibit the expression of MAGEB5 which had a non-significant variation per the relationship model. Based on the null hypothesis, the lesser the prediction percentage per immune variant the more effective it is against targeted epitope peptides predicted for the protein variants of HHLA2 and MAGEB5 genes. The variant prediction frequency for HLA-C2 was higher than that of HLA-C9 though they showed a similar epitope peptide variation for HHLA2 and MAGEB5, suggesting that, the possible contribution of HHLA1 and HHLA3 could affect immune variant efficiency. Therefore, the lowest prediction percentage in the data should be considered a better target for immune variant immunotherapy design. The modelled relationship of HLA-DRB5 = HHLA2 + MAGEB5 + C showed no statistically significant value for any dependent variable and the immune variants in consideration, which should suggest that the immune impact by HLA class II molecules works independently of the interaction between HHLA2 and MAGEB5. This was further confirmed by the fact that, there was no positive correlation between HHLA2 and MAGEB5. Therefore, in designing target immune epitopes for therapy using MHC HLA class II molecules, epitope peptides for HHLA2 and MAGEB5 should be equally considered as potential targets and independent from one another. The relationship type observed for MHC class I (HLA-C) immune variants targeting HHLA2 epitope peptides for the control of MAGEB5 brings into mind gene drivers in protein interaction biological networks wherein targeting the driver controls the network and prevents the biological driven process. LCK gene is a lymphoid-specific phosphor tyrosine kinase (PTK) with role in the maturation of T-cells and also transduction of signals from the T-cell receptor upon binding to an antigen. Genetic targeting of LCK gene in mice showed improper thymocyte maturation to CD4 and CD8 T-cell phenotypes and also compromising the TCR signaling pathway [37]. From these studies HHLA2 can be likened to the role of LCK in a functional network involving MAGEB5 and other related proteins, wherein disrupting the function of HHLA2, inhibits the successful expression of MAGEB5, hence preventing any viral related manifestations resulting from HHLA2 and MAGEB5 co-expression. These results demonstrate a genetic disease interaction network which could be driven by HHLA2 and epitope peptide targeting with the right immune variant could be a good clinical solution to be considered.

## Conclusion

This study focused on understanding how key expressed genes in a cancer related viral disease could be considered for immune targeted studies against epitope peptides of targeted genes. We suggest that Laboratory testing of these predicted variants are needed for molecular affirmation. HHLA 1, 2 & 3 and MAGEB5 were the most expressed in a previous study and we could see specific positive correlation patterns between HHLA2 and MAGEB5. Both genes are expressed in case of a tumor and linking them to viral data associates their co-expression to viral related cancers. This study is the first to model targeted specific immune variants and their related antigenic epitopes with aim to understand how to control gene expression by targeting important genetic players in a protein biological disease network. The variation of HHLA2 epitope peptides happened to be statistically significant with HLA class I (HLA-C) immune variants considered in this study and their percentage prediction scores are a great way of evaluating their effect on the model. This analysis translates into a targeted immune-antigenic application study within a population wherein the immune variant distribution within the community needs to be deciphered and used as a basis to target disease related genes. Viruses are involved in several cancer types and drive several kinds of downstream genomic expressions based on their portal of entry within the cell which leads to chronic viral diseases like HIV and hepatitis C and or cancers like Hodgkin lymphoma. Additional analysis and understanding of these downstream reactions is a key to properly inhibiting viral disease effects within humans and populations. Further analysis of the expression and genomic interaction of identified epitope peptides between HHLA2 and MAGEB5 in relation to specific immune variants provides a good basis of therapeutic approaches to inhibit this reaction and prevent viral effects related to chronic diseases and cancers.

## Supplementary Information

**Additional file 1.**

## Data Availability

Dataset 1 MAGE-virus samples identified (*n* = 10) on GEO profile database; http://article.scholarena.com/datasets/Dataset_1.xls The datasets generated and/or analyzed during the current study are available in [23]. The datasets generated and analyzed during the current study were obtained from the GEO (RRID: SCR_005012) repository, and specifically GEO Profiles database (www.ncbi.nlm.nih.gov/geoprofiles/) with the Accession numbers: GDS5093, GDS4424, GDS5614, GDS5613, GDS2606, GDS4238, GDS2023, GDS3489, GDS2676, GDS4669. The following datasets below were generated and analyzed in a previous study [23] and also used during the current study, but are not publicly available because they have not yet been deposited in a public repository but are available from the corresponding author or the referenced article above [23] as indicated. Dataset 2 MAGE-virus gene values per sample retrieved (n = 10) from GEO profile database; http://article.scholarena.com/datasets/Dataset_2.xls Dataset 3 MAGE-virus normalized gene RMEAN values (*n* = 19,688) across samples; http://article.scholarena.com/datasets/Dataset_3.xls Dataset 4 MAGE-virus immune related genes (*n* = 69) across samples; http://article.scholarena.com/datasets/Dataset_4.xls Dataset 5 MAGE-virus most variable genes (*n* = 200) across samples; http://article.scholarena.com/datasets/Dataset_5.xls Dataset 6 MAGE-virus Gene Ontology protein interaction biological process data; http://article.scholarena.com/datasets/Dataset_6.xls
