# Supplementary material for: Targeted immune epitope prediction to HHLA2 and MAGEB5 protein variants as therapeutic approach to related viral diseases
Source: BMC Immunol. 2021 Jul 28;22:49. doi: 10.1186/s12865-021-00440-w (PMC8316541; doi:10.1186/s12865-021-00440-w)
Supplement: Supplementary file 1 — Additional file 1. [file 12865_2021_440_MOESM1_ESM.docx]

**Supplementary Material**

Targeted immune epitope prediction to HHLA2 and MAGEB5 protein variants as therapeutic approach to related viral diseases.

**Authors Information**

**Daniel A. Achinko, PhD ^1,2 *^**

Anton Dormer, MD ^1,2^

Mahesh Narayanan, MSc ^1,2^

Elton F. Norman ^1,2^

1. PepVax, Inc.10411 Motor City Drive Bethesda, MD 20817, USA.

2. Howard University Hospital, College of Medicine, 2041 Georgia Avenue, Washington DC, 20060.

Corresponding author email: [da4815@gmail.com](mailto:da4815@gmail.com); daniel@pepvax.co

**Availability of data and materials**

Dataset 1: MAGE-virus samples identified (n=10) on GEO profile database; <http://article.scholarena.com/datasets/Dataset_1.xls>

The datasets generated and/or analyzed during the current study are available in [23].

The datasets generated and analyzed during the current study were obtained from the GEO (RRID: SCR_005012) repository, and specifically GEO Profiles database ([www.ncbi.nlm.nih.gov/geoprofiles](http://www.ncbi.nlm.nih.gov/geoprofiles)/) with the Accession numbers: GDS5093, GDS4424, GDS5614, GDS5613, GDS2606, GDS4238, GDS2023, GDS3489, GDS2676, GDS4669.

The following datasets below generated and analyzed during the current study are not publicly available because they have not yet been deposited in a public repository but are available from the corresponding author or the referenced article above [23] as .

Dataset 2: MAGE-virus gene values per sample retrieved (n=10) from GEO profile database; <http://article.scholarena.com/datasets/Dataset_2.xls>

Dataset 3: MAGE-virus normalized gene RMEAN values (n=19688) across samples; <http://article.scholarena.com/datasets/Dataset_3.xls>

Dataset 4: MAGE-virus immune related genes (n=69) across samples; <http://article.scholarena.com/datasets/Dataset_4.xls>

Dataset 5: MAGE-virus most variable genes (n=200) across samples; <http://article.scholarena.com/datasets/Dataset_5.xls>

Dataset 6: MAGE-virus Gene Ontology protein interaction biological process data; <http://article.scholarena.com/datasets/Dataset_6.xls>
